# The Modified Cupid's Bow Lips: A Dominant Aesthetic Trend in Lip Augmentation—A Clinical and Statistical Analysis

**DOI:** 10.1111/jocd.70855

**Published:** 2026-04-07

**Authors:** Dong Wang, Yaqin Xu, Zhiqiang Yang

**Affiliations:** ^1^ Yamei Medical Aesthetic Clinic Guangzhou China; ^2^ Only Beauty Dermatology Medical Aesthetic Clinic Chengdu China

**Keywords:** facial aesthetics, hyaluronic acid filler, injection technique, lip augmentation, modified cupid's bow lips, morphometric analysis

## Abstract

**Background:**

Contemporary lip augmentation has evolved from simple volumization techniques to more intricate contour‐specific enhancements. Among these advancements, the Modified Cupid's bow lips, characterized by their distinctive Cupid's bow shape and vertical projection, have gained significant popularity as a prominent aesthetic trend.

**Aims:**

This study aims to evaluate the prevalence of patient preference for the Modified Cupid's bow lips contour and to propose a systematic approach for its clinical implementation based on retrospective analysis.

**Methods:**

A retrospective analysis was conducted, including 67 consecutive female patients seeking primary lip augmentation. Demographic information and preferences regarding lip contours were statistically analyzed using chi‐square tests with age stratification. Anthropometric measurements of labial tubercle area and upper lip angles were performed using Image Pro Plus software for quantitative assessment.

**Results:**

This analysis revealed that 42 patients (62.7%) preferred Modified Cupid's bow lips, a significantly higher proportion compared to the 25 patients (37.3%) who favored natural lips (*χ* ^2^ = 4.31, *p* = 0.038). In addition, a significant correlation was found between age group and lip preference (*χ* ^2^ = 8.74, *p* = 0.033), with the highest preference for Modified Cupid's bow lips (73.9%) that was observed in the 31–40 year age group. Morphometric analysis demonstrated statistically significant post‐treatment increases in labial tubercle area (228.5 ± 169.6 mm^2^ vs. 297.0 ± 157.3 mm^2^, *p* = 0.039) and alterations in key angular measurements (Angle *γ*, *δ*, *ε*; *p* < 0.05), confirming enhanced central projection, vertical height, and contour definition.

**Conclusion:**

The Modified Cupid's bow lips represent a dominant aesthetic preference and necessitate specialized injection techniques and an understanding of anatomy for optimal results. Objective morphometric changes validate the efficacy of the technique in achieving defined lip contours.

## 1 Introduction

The ideal paradigm of lip aesthetics has undergone a significant transformation in recent years [1]. Previously, the primary focus was on enhancing volume; however, current trends emphasize structural definition, proportional balance, and contouring that caters to diverse ethnicities. This shift reflects advancements in technical capabilities and evolving cultural preferences towards more personalized aesthetic outcomes [1, 2, 3].

In everyday medical aesthetic practice for lip augmentation, a multidimensional assessment is routinely conducted. This comprehensive evaluation encompasses anatomical, age‐related, and aesthetic analyses. The anatomical assessment evaluates the structural integrity, proportions, and symmetry of the lip subunits. The aging evaluation examines volume loss within these subunits, the clarity and fluency of the vermilion border, and the three‐dimensional relationship between the red lip (vermilion) and the surrounding cutaneous lip [1]. The aesthetic analysis considers the overall lip morphology and examines how the details of the subunits add to overall facial harmony [3].

Both practitioners and patients place significant emphasis on lip shape morphology [1]. In our clinic, we have observed a notable increase in female patients requesting the Modified Cupid's bow lip contour, highlighting a growing emphasis on refined treatment details. The Modified Cupid's bow lip contour has recently gained substantial popularity in our medical aesthetic clinic. This contour is defined by a prominent Cupid's bow, a full upper tubercle, elevated lip peaks, and increased vertical projection. It represents a meticulous move towards precision enhancement that maintains natural balance. Despite its widespread popularity in clinical practice, there is an absence of robust statistical analysis regarding this preference and standardized protocols for achieving it in the literature.

**FIGURE 1:**
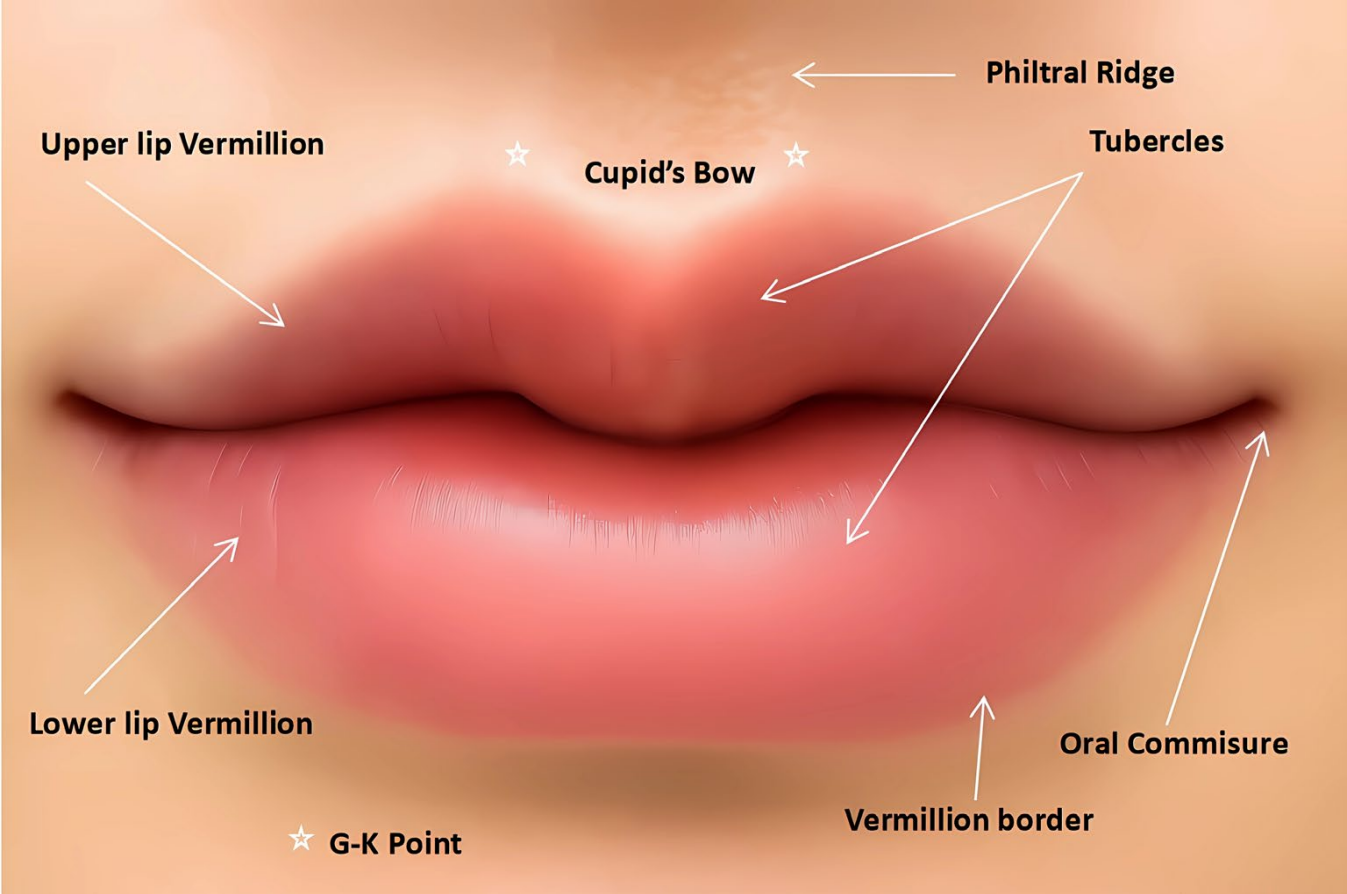
Diagram of modified Cupid's bow lip anatomy.

This study aims to quantitatively assess the patient‐driven demand for the Modified Cupid's bow lips (Figure 1).

## 2 Patients and Methods

### 2.1 Study Design and Patient Selection

A retrospective analysis was conducted, including 67 consecutive female patients seeking primary lip augmentation between January 2025 and October 2025. The inclusion criteria were as follows: comprehensive documentation of demographic data and lip shape preference during consultation. Patients aged 18 years or above with no prior instances of severe adverse reactions to dermal fillers were enrolled. Exclusion criteria included active infection at the treatment site, known allergies to any components of the hyaluronic acid fillers, a history of permanent filler use in the lips, or the presence of any systemic condition that would preclude the safe administration of cosmetic injectables.

### 2.2 Treatment Protocol

A pre‐treatment consultation was conducted for each patient, which included a systematic evaluation of lip morphology. During the initial consultation stage, we provide a standardized visual reference gallery for the beauty seekers to clarify their aesthetic preferences. The gallery includes pre‐approved photos of celebrities' or models' lips, categorized into styles such as “Improved Cupid's Bow” or “Natural”, clearly demonstrating key differences in contour, fullness, and symmetry. Through interactive methods, the shape and fullness can be adjusted specifically, visually presenting different effects such as “improvement” (e.g., increasing the fullness of the middle part of the lips) and “naturalness” (e.g., enhancing the sense of balance). The beauty seekers can confirm through two‐dimensional pre‐ and post‐operative comparison images. In addition, Image Pro Plus software will be used for angle measurement and analysis to provide objective data support. Based on this assessment, a standardized treatment protocol was established and standardized photographic documentation was taken pre‐ and post‐procedure for all cases.

### 2.3 Injection Techniques and Materials

The Modified Cupid's bow lip augmentation procedure in this practice follows a systematic methodology characterized by a lateral‐to‐medial, superior‐to‐inferior direction with incremental volume deposition. The technique is tailored to emphasize the lip tubercle and create a subtle elevation of the commissure according to the individual aesthetic goals.

The lips were divided into three anatomical zones for precise injection:

- Upper lip: lateral segments (UL^1^), medial segments (UL^2^), and tubercle (UT^3^)
- Lower lip: lateral segments (LL^1^) and medial tubercle (LT^2^)
- Oral commissure (OM^1^)

**FIGURE 2:**
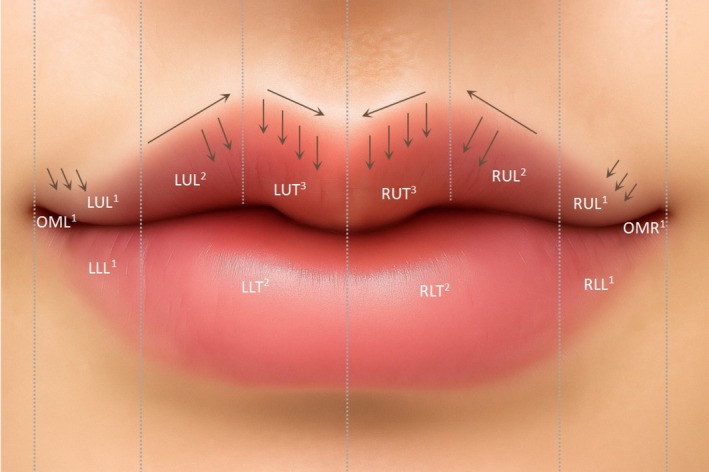
Modified Cupid's bow lip injection point.

*Note:* LL = Lower Lip, LT = Lower Tubercle, OM = Oral Commissure, UL = Upper Lip, UT = Upper Tubercle.

All injections were performed using lip‐specific fillers and a 30‐gauge, 13‐mm needle. Each treatment was customized according to the patient's individual anatomy. The treatment adhered to the “minimal effective dose” principle to achieve optimal aesthetic outcomes. Injections of filler (lower cohesivity and lower G′) were administered in the deep submucosal plane following the given sequence: upper lip (UL^1^ → UL^2^ → UT^3^), lower lip (LL^1^ → LT^2^), and finally the oral commissure (OM^1^). Injection targets emphasize three zones (Figure 2): the central lip tubercle for projection and “M” contour; the vermilion body junction area with upper lip tubercle for seamless transitions; and the vermilion border for sharp definition and symmetry. This approach balances structural support with aesthetic refinement. While maintaining anatomical harmony, this structured approach ensures optimal contour definition and natural‐looking volume enhancement. Injection dose in each zone was lower than 0.1–0.15 mL at UL^1^, UL^2^, UT^3^.

### 2.4 Data Collection and Statistical Analysis

Data extraction encompassed patient age and documented preferences for either “Modified Cupid's bow lips” or “natural lips.” SPSS version 22.0 (IBM Corp., Armonk, NY) was used to perform statistical analysis. Patients were categorized into four age groups for comparative purposes. Pearson's chi‐square test was utilized to evaluate the associations between age and lip preference. Statistical significance was set at *p* < 0.05.

## 3 Results

### 3.1 Demographic Characteristics and Preference Distribution

The cohort exhibited a notable preference for Modified Cupid's bow lips, with 62.7% (42/67) of patients selecting this option compared to 37.3% (25/67) who preferred natural lips. This distribution was statistically significant (*χ* ^2^ = 4.31, *p* = 0.038). Table 1 presents detailed results.

**TABLE 1:** Preference of lip shape in the patient cohort.

Characteristic	Total (*N* = 67)	*p*
Lip shape preference		
Modified Cupid's bow lips	42 (62.7%)	0.038^a^
Natural	25 (37.3%)	

^a^ Data indicates *p* < 0.05, representing statistical significance.

### 3.2 Age‐Related Preference Patterns

Analysis demonstrated significant variation in preference across age groups (*χ* ^2^ = 8.74, *p* = 0.033). The strongest preference for Modified Cupid's bow lips was seen in patients aged 31–40 years (73.9%), while a greater preference for natural lips (66.7%) was observed in the ≤ 25 years group. Table 2 presents detailed results.

**TABLE 2:** Distribution of the shape preferences of lips by age group.

Age group (years)	Total, *n*	Modified Cupid's bow lips, *n* (%)	Natural lip, *n* (%)
≤ 25	15	5 (33.3)	10 (66.7)
26–30	23	15 (65.2)	8 (34.8)
31–40	23	17 (73.9)*	6 (26.1)
≥ 41	6	5 (83.3)	1 (16.7)
Total	67	42 (62.7)	25 (37.3)

^*^ Data are presented as the number of patients (*n*) and percentage (%). Overall comparison across age groups was performed using the Chi‐square test, *χ* ^2^ = 8.74, *p* = 0.033.

### 3.3 Anthropometric Results of the Modified Cupid's Bow Lips Group

**FIGURE 3:**
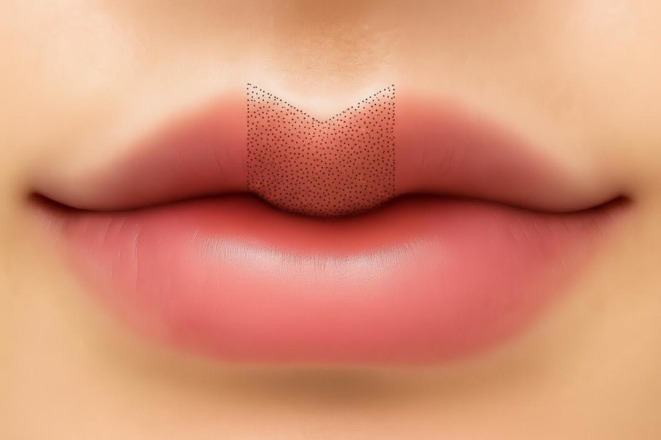
Upper lip tubercle area.

**FIGURE 4:**
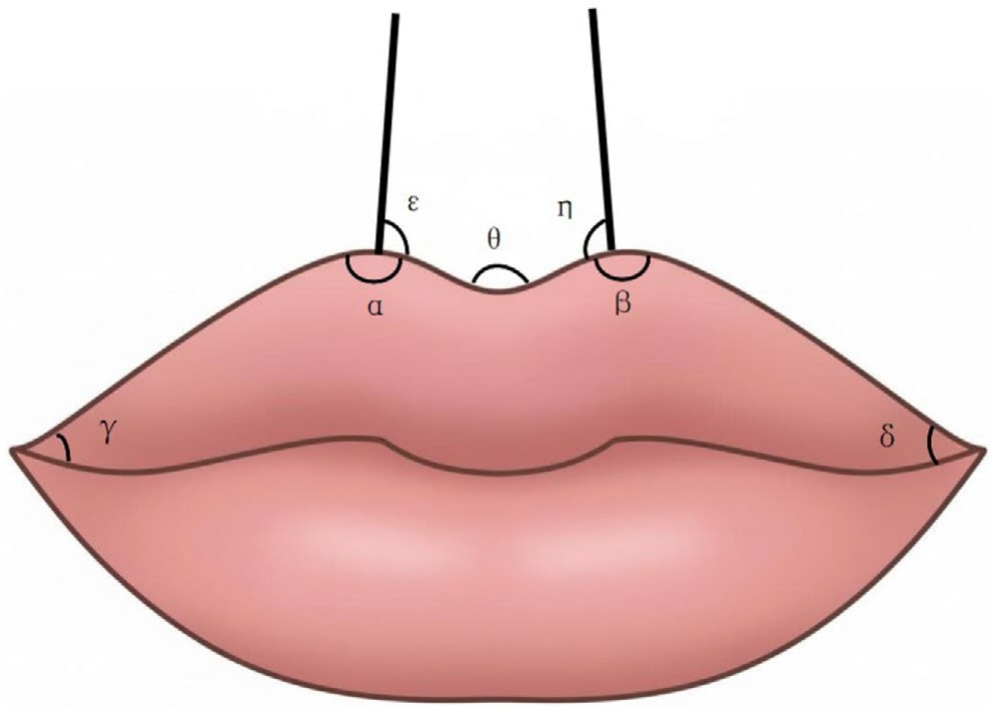
Upper lip angular measurements.

Statistical analyses were conducted on labial morphology data from the 26–40 years old cohort, who represented the primary demographic opting for the modified Cupid's bow lips procedure. Standardized pre‐ and post‐procedure photographs of the treatment group served as the basis for quantitative assessment. Using Image Pro Plus software, the area of the labial tubercle (Figure 3) and the angular measurements of the upper lip (Figure 4) were obtained for subsequent correlation analysis. The area of the lip tubercle region is defined as follows: a vertical line is drawn downward from the bilateral G–K points to the vermilion border. The region enclosed inward by this line, together with the Cupid's bow and the vermilion border of the lip, constitutes the measured area (as illustrated).

The morphometric results demonstrated statistically significant alterations in labial morphology following treatment (Table 3). The area of the labial tubercle showed a significant increase (228.5 ± 169.6 mm^2^ vs. 297.0 ± 157.3 mm^2^, *p* = 0.039), objectively confirming that filler treatment effectively augmented the central projection and volume of the upper lip. This enhancement is crucial for achieving the hallmark “full tubercle” characteristic of the modified Cupid's bow contour.

**TABLE 3:** Comparison of labial angle results of the modified Cupid's bow lips group before and after treatment (*n* = 32).

Measurement	Before (mean ± SD)	After (mean ± SD)	*p*
Upper lip tubercle area (mm^**2**^)	228.5 ± 169.6	297.0 ± 157.3	0.039^a^
Upper lip angle *α*(°)	126.5 ± 14.0	120.8 ± 5.5	0.136
Upper lip angle *β*(°)	120.5 ± 10.9	116.6 ± 7.7	0.186
Upper lip angle *γ*(°)	23.8 ± 4.2	27.2 ± 5.0	0.034^a^
Upper lip angle *δ*(°)	24.3 ± 4.5	27.3 ± 4.8	0.048^a^
Upper lip angle *ε*(°)	109.1 ± 10.7	101.8 ± 9.2	0.043^a^
Upper lip angle *η*(°)	97.9 ± 12.6	94.8 ± 8.9	0.392
Upper lip angle *θ*(°)	119.2 ± 18.2	128.2 ± 8.9	0.088

^a^ Data indicates *p* < 0.05, representing statistical significance.

Angular measurements further revealed refined contour changes. Angle γ and Angle δ, representing the lateral slope of the upper lip, increased significantly (from 23.8° ± 4.2° to 27.2° ± 5.0°, *p* = 0.034; and from 24.3° ± 4.5° to 27.3° ± 4.8°, *p* = 0.048, respectively). This change indicates an improvement in the vertical height and projection of the lateral upper lip (from the peak of the Cupid's bow to the commissure), contributing to a more defined and three‐dimensional lip arch. Concurrently, Angle ε (reflecting the nasolabial relationship) decreased significantly (109.1° ± 10.7° vs. 101.8° ± 9.2°, *p* = 0.043), suggesting the upper lip gained better vertical support and slight anterior rotation, thereby enhancing its upward tilt and youthful appearance.

Notably, Angle *α* and Angle *β*, which directly characterize the acuteness of the Cupid's bow apex, showed a decreasing trend post‐treatment (from 126.5° ± 14.0° to 120.8° ± 5.5°, and from 120.5° ± 10.9° to 116.6° ± 7.7°, respectively), though these changes did not reach statistical significance (*p* > 0.05).

### 3.4 Different Age Group Lip Cases

**FIGURE 5:**
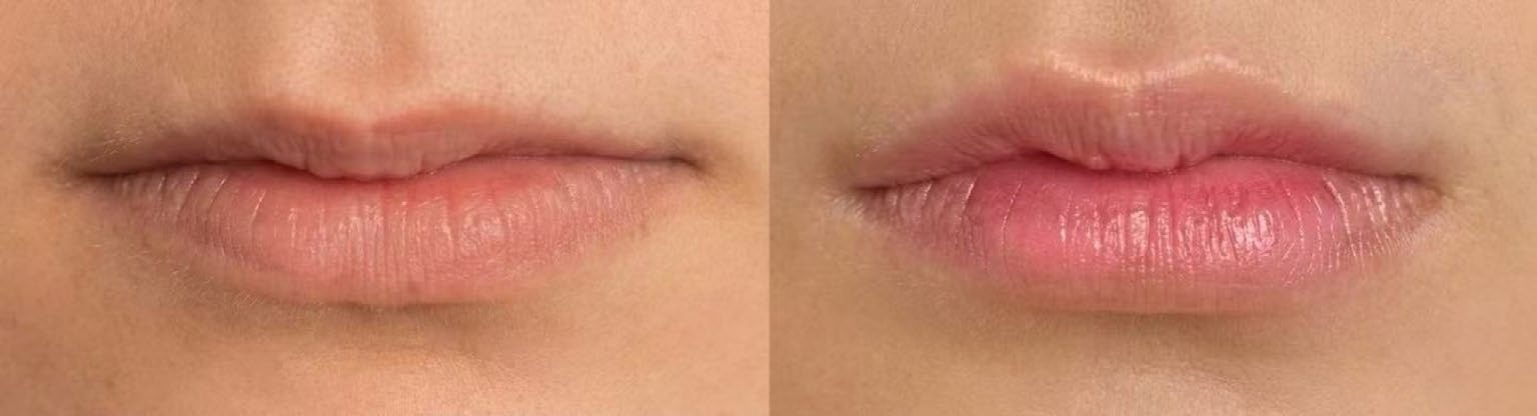
Case 1: Chinese female. A 26‐year‐old Chinese female treated using 1 mL of hyaluronic acid filler. Her preinjection (left) and postinjection (right) clinical images are shown.

**FIGURE 6:**
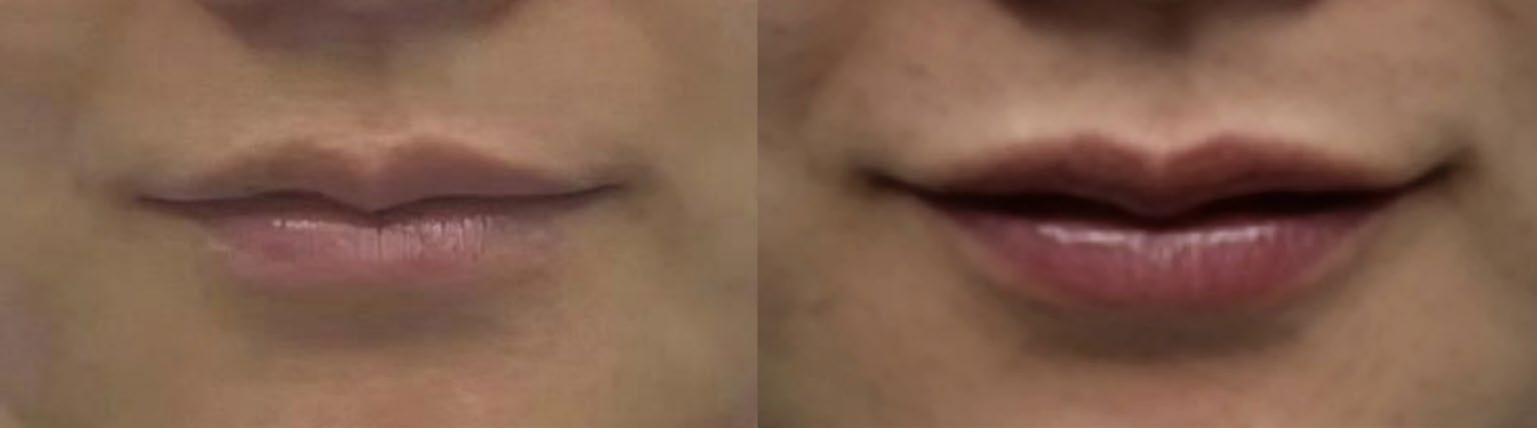
Case 2: Chinese female. A 38‐year‐old Chinese female treated using 1 mL of hyaluronic acid filler. Her preinjection (left) and postinjection (right) clinical images are shown.

**FIGURE 7:**
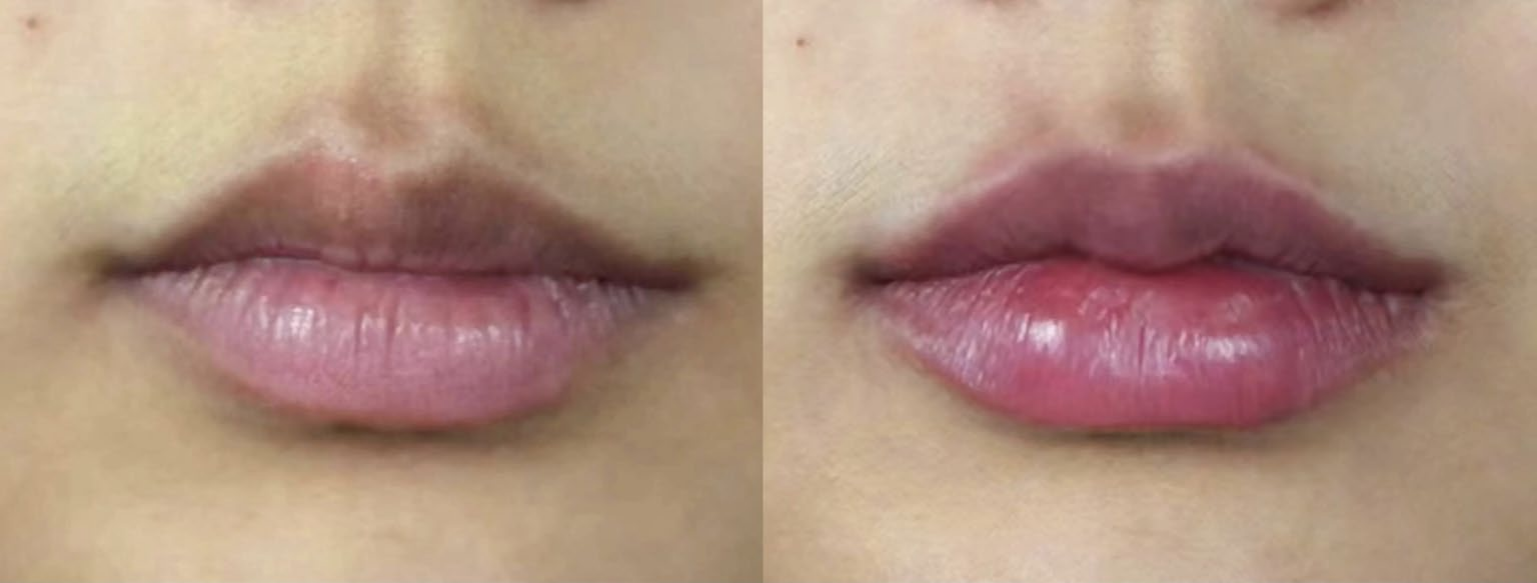
Case 3: Chinese female. A 25‐year‐old Chinese female treated using 1 mL of hyaluronic acid filler. Her preinjection (left) and postinjection (right) clinical images are shown.

These three cases illustrate how identical treatments yield distinct outcomes based on age‐specific anatomical needs (Figures 5, 6, 7). In the young adult cohort (Cases 1 and 3, ages 25–26), where tissue elasticity remains high, the filler primarily served an aesthetic contouring role, enhancing Cupid's bow definition and projection to create a fuller silhouette. Conversely, for the mature patient (Case 2, age 38), the same volume addressed age‐related volume depletion and vermilion border flattening, focusing on structural restoration to reverse signs of aging. This comparison underscores that while dosage was constant, the therapeutic intent shifted from beautification in youth to volumetric rehabilitation in maturity, highlighting the necessity of tailoring injection strategies to physiological age.

## 4 Discussion

This study utilizes statistical analysis to confirm that Modified Cupid's bow lips have become the predominant aesthetic preference, with 62.7% of individuals seeking lip augmentation favoring this style over natural lips. This observation underscores a notable shift in demand, reflecting the current global trend in aesthetic medicine that emphasizes contour and structural definition. Moreover, the study reveals the underlying factors influencing this trend, particularly the significant role of age in shaping aesthetic preferences.

Our data indicate a significant age‐dependent variation in these preferences (*p* = 0.033), with a pronounced favoring of Modified Cupid's bow lips in mature women (> 26 years). This trend likely mirrors the distinct aesthetic priorities and anatomical needs that vary across different age groups.

Younger individuals aged 25 or younger exhibited a strong preference for natural‐looking lips, with 66.7% of individuals favoring this aesthetic. This age group primarily aims for an effect that underscores their innate beauty rather than making drastic changes to the shape of their lips; thus, the main objective is to achieve subtle volume restoration and enhance natural lip characteristics.

In contrast, individuals above 31 years of age exhibited a growing preference for Modified Cupid's bow lips. This trend can be attributed to several factors. Firstly, age‐related anatomical changes become more noticeable, including blurring of the vermilion border, volume loss in the lip tubercle, and mild ptosis of the oral commissure [4, 5]. The Modified Cupid's bow lips injection technique effectively addresses these changes through precise contour definition and enhancement of vertical height, thus achieving effective structural rejuvenation rather than just aesthetic improvement [6]. Secondly, individuals in this age group typically have more experience with aesthetic treatments and greater financial resources, seeking more refined, designed, and effective treatments [7]. The “precision aesthetics” represented by the Modified Cupid's bow lips align with this demand.

When administering injections to create a modified Cupid's bow, the practitioner must pay careful attention to the subtle transitions between lip subunits, especially at the junction between the upper vermilion body and the tubercle [8]. In addition, practitioners should also make adjustments based on the patient's aesthetic preferences regarding overall lip fullness, the relationship between the upper and lower tubercles, and the desired level of commissure elevation [9, 10].

This study establishes a quantitative link between the subjective aesthetic goals of the modified Cupid's bow lip contour and objective morphological changes through measurement. As shown in Table 3, the significant increase in labial tubercle area directly corresponds to the clinically observed central volume enhancement, which serves as the foundation of this lip shape. The increase in Angle γ and Angle δ geometrically confirms the enhancement of lip peak height and lateral contour, explaining the postoperative visual effect of a more defined and three‐dimensional Cupid's bow. The decrease in Angle ε (sharpening of the nasolabial angle) signifies improved vertical support and anterior rotation of the upper lip, which is closely associated with the clinically desired “subtle upward tilt” of the upper lip and the correction of age‐related flattening. These data translate clinical experience into quantifiable evidence, providing an objective standard for treatment goals and outcome assessment.

Collectively, these objective measurement data substantiate that the modified Cupid's bow lip injection technique can systematically alter the geometric parameters of specific lip subunits, ultimately achieving the comprehensive aesthetic outcome of a prominent tubercle, defined lip peaks, and enhanced upper lip projection.

The standardized injection protocol, namely zonal and sequential injection, proposed in this study holds significant clinical value. It provides clinicians, particularly those early in their learning curve, with a safe, reproducible, and logically clear operational pathway. This framework minimizes operational uncertainty by deconstructing the complex lip anatomy into specific subunits (UL^1^, UL^2^, UT^3^, etc.) and prescribing an injection sequence. It helps in the improvement of consistency in treatment outcomes and reduces the risk of complications such as asymmetry.

This study has several limitations. The generalizability of the findings is limited by their retrospective design and single‐center data source. Although the total sample size (*N* = 67) was sufficient to show the primary trend, the sample size was relatively small in the ≥ 41 age group, and results for this group need further validation in larger cohorts.

## 5 Conclusion

The Modified Cupid's bow lips represent a significant and dominant trend in modern aesthetic practice. The statistical analysis confirms strong patient preference for this defined lip contour, particularly among specific age demographics [11]. Successful execution of this contour requires a comprehensive understanding of lip anatomy, strategic product selection, and meticulous injection technique. Thus, the standardized protocol presented herein offers clinicians a reliable framework for fulfilling patient expectations while maintaining natural facial harmony.

## Author Contributions

**Dong Wang:** conceptualization, methodology, formal analysis, investigation, data curation, writing – original draft. **Zhiqiang Yang:** supervision, formal analysis, writing – review and editing. **Yaqin Xu:** methodology, data curation, writing – review and editing.

## Funding

The authors have nothing to report.

## Consent

Informed consent was obtained from all participants, and the study was approved by Academic Ethics Committee of Yamei Aesthetic Medical Center, Guangzhou.

## Conflicts of Interest

The authors declare no conflicts of interest.

## Data Availability

The datasets generated and/or analyzed during the current study are available from the corresponding author on reasonable request.
